# Follicular Dynamics during Estrous Cycle of Pubertal, Mature and Postpartum Crossbred (Nili Ravi × Jianghan) Buffaloes

**DOI:** 10.3390/ani12091208

**Published:** 2022-05-07

**Authors:** Adili Abulaiti, Umair Riaz, Zahid Naseer, Zulfiqar Ahmed, Guohua Hua, Liguo Yang

**Affiliations:** 1 Key Laboratory of Animal Genetics, Breeding and Reproduction, College of Animal Science and Technology, Ministry of Education, Huazhong Agricultural University, Wuhan 430070, China; adiliabulaiti@webmail.hzau.edu.cn (A.A.); umair.riaz@iub.edu.pk (U.R.); zlfqr_abbasi@yahoo.com (Z.A.); 2 International Joint Research Center for Animal Genetics, Breeding and Reproduction, Wuhan 430070, China; 3 Hubei Province’s Engineering Research Center in Buffalo Breeding & Products, Wuhan 430070, China; 4 Key Laboratory of Smart Farming for Agricultural Animals, Wuhan 430070, China; 5 Department of Theriogenology, Faculty of Veterinary and Animal Sciences, The Islamia University of Bahawalpur, Bahawalpur 63100, Pakistan; 6 Theriogenology Section, Department of Clinical Studies, Faculty of Veterinary and Animal Sciences, Pir Mehr Ali Shah Arid Agriculture University, Rawalpindi 46000, Pakistan; zahidnaseer@uaar.edu.pk

**Keywords:** Nili Ravi-Jianghan crossbred buffalo, follicular dynamics, estrous cycle, ultrasonography

## Abstract

**Simple Summary:**

The objective of this study was to observe the follicle dynamics and its relationship with different physiological (pubertal, sexual mature and postpartum) conditions of buffaloes. These data could be beneficial in establishing best reproductive model for future studies and enhancing the efficiency of advanced reproductive technologies (ARTs) in buffalo industry. The present study describes the estrous cycle characteristics (follicular wave pattern, ovulation, follicle growth, regression or atresia) in Chinese crossbred buffaloes during pubertal, sexual maturity and postpartum stages. This study would be helpful to provide the theoretical insight to overcome the poor reproductive performance by employing the ARTs to enhance fertility in crossbred buffaloes.

**Abstract:**

The follicular dynamics is used as a reliable indicator for reproductive management in livestock. However, the follicular dynamics (follicle wave emergence, estrus cycle length, diameter of dominant follicle, follicular growth and atretic phases) during the estrous cycle of crossbred (Nili Ravi-Jianghan) buffalo is still unexplored. Therefore, the present study aimed to observe the follicular dynamics in estrous cycle of crossbred buffaloes at different physiological stages (pubertal; *n* = 28, sexual mature; *n* = 22 and postpartum; *n* = 18). In the present study, the follicular dynamics were ultrasonically examined at 12 h intervals throughout an estrous cycle during the breeding season. The results indicate that about 86.76% (59/68) crossbred buffaloes, irrespective of physiological stage, exhibited two follicular waves in estrous cycle with an average estrus cycle length was 20.7 ± 0.4 days. The estrus cycle length was significantly shorter (*p* < 0.05) in pubertal buffaloes (19.4 ± 0.4 days) compared with sexual mature (21.5 ± 0.3 days) and postpartum (21.9 ± 0.4 days) buffaloes. The first follicular wave emerged on same day during one- (pubertal vs. postpartum), two- (pubertal vs. mature vs. postpartum) or three-wave (mature vs. postpartum) estrous cycle buffaloes. The maximum diameter of dominant follicle (DF) in pubertal, sexually mature and postpartum crossbred buffaloes was 9.6 ± 2.0 mm, 10.6 ± 0.5 mm and 12.6 ± 0.7 mm with growth rate of 1.08 ± 0.04 mm/day, 0.92 ± 0.04 mm/day, and 0.9 ± 0.07 mm/day, respectively. In conclusion, similar to other buffalo breeds, Nili Ravi-Jianghan crossbred buffaloes showed the two-wave follicular pattern dominantly with an average duration of ~20 days estrous cycle. The observed follicular dynamics can be used as a reliable indicator for synchronization and fixed-time artificial insemination (FTAI) programs to improve the fertility of crossbred buffaloes.

## 1. Introduction

The majority of the Chinese indigenous buffalo population (99%) is composed of swamp types which have poor milk production compared with river-type buffaloes. Crossbreeding of swamp and river buffalo resulted in a crossbred buffalo breed [1], which has a higher milk production capacity compared with the indigenous swamp type. The Binglangjiang buffalo is the only river-type buffalo that was mainly distributed for more than two centuries in the Binglang river basin of Tengchong County, Yunnan Province, China. This buffalo breed remained a valuable genetic resource for the development of the milk buffalo industry in China through crossbreeding of swamp and riverine buffaloes [2,3,4]. To enhance the productivity of local Chinese swamp buffalo, the exotic buffalo breeds (Murrah and Nili-Ravi) were also introduced. The Nili-Ravi × Jianghan crossbred buffalo is one of the major breeds in Central China, and it provides the better production levels compared with other crossbred breeds. However, this crossbred buffalo breed (Nili-Ravi × Jianghan) compromised reproductive performance similar to pure swamp or riverine breeds. This breed modification introduced new challenges to understand the basic follicular dynamics pattern in the crossbred buffaloes.

Follicular dynamics are the continuous process of growth and regression of a group of antral follicles, one of which develops until it reaches the pre-ovulatory stage [5]; therefore, an understanding of follicular development and regression is essential in order to ameliorate the existing techniques of estrus control techniques [6,7,8]. The success of reproductive control programs is closely related to an understanding of ovarian follicular dynamics [9]. Bovine follicular dynamics have been extensively studied at different phases of the estrous cycle [10], pregnancy [11], pubertal [12] and postpartum stage [13] which allowed successfully implementation of the assisted reproductive technologies (ARTs). Similar efforts have also been made in pure buffalo breeds but the outcomes of ARTs are variable [14] due to different estrus physiology of buffalo compared to cattle [15].

Along others influencing factors in breeding type, the age or physiological status (puberty, maturity or postpartum) of buffalo is an important element for reproductive efficiency. The low bodyweight of buffalo heifers also delays the pubertal age in response to unavailability of nutrients under semi-intensive rearing systems [16]. It has been observed that Chinese crossbred buffalo heifers attain the puberty at 22 months of age [17], but underdevelopment of reproductive tract does not provide optimum breeding time prior to 25 months of age. In the case of postpartum Chinese crossbred buffalo, ovarian activity is resumed within 40 days of calving, but estrus response is very low along the first and second ovulations [17]. In addition, the application of FTAI protocols is variable in heifers and postpartum buffaloes with respect to the season of the year [18]. Previously, several aspects of the estrous cycle and follicular dynamics were studied in Nili-Ravi [19,20], Murrah [21,22,23] and Mediterranean [24] buffaloes. However, the exact information regarding follicular dynamics during estrous cycle is not well understood in crossbred buffaloes of different physiological stages [25]. For proper application of ARTs Chinese crossbred buffaloes, the monitoring of ovarian dynamics at pubertal, mature and postpartum stages could be potential aspects to overcome the above-mentioned concerns in buffaloes at different physiological stages. Therefore, the present study was designed to observe the ovarian follicular dynamics during the estrous cycle of Chinese crossbred (Nili-Ravi × Jianghan) buffaloes at pubertal, sexually mature and postpartum stages.

## 2. Material and Methods

### 2.1. Ethics Statement

The present study was approved by the Animal Welfare and Ethical Committee, Huazhong Agriculture University, People’s Republic of China (Approval ID: HZAUBU-2017-002). All experimental protocols were followed according to guidelines proposed by the Committee of Animal Research Institute, Huazhong Agricultural University, China.

### 2.2. Experimental Animals

The crossbred buffaloes (Nili-Ravi × Jianghan; *n* = 68) were selected during breeding season (12 September 2017 to 11 March 2018) at a buffalo farm located in Hubei Jinniu Co., Ltd., Hubei province, China. The buffaloes were healthy with a moderate body condition score (BCS = 3.4 ± 0.6) without any palpable abnormality in the reproductive tracts. The selected buffaloes were divided in three groups based on different physiologic status (pubertal, mature and postpartum). The first group includes the pubertal buffaloes (*n* = 28) without any history of estrus behavior having an average body weight (BW) of 308.2 ± 38.6 kg at age of 1.7 ± 0.2 years. The pubertal group of buffalo was monitored ultrasonographically on a daily basis for the occurrence of ovulation during the observational period, and pubertal buffaloes were characterized as pubertal when the first ovulation was recorded. The second group includes sexually mature crossbred buffaloes (*n* = 22) having BW of 433.0 ± 72.1 kg and age of 3.2 ± 0.5 years with a cyclic history. In postpartum group, the multiparous postpartum buffaloes (*n* = 18) with BW of 588.1 ± 89.5 kg and age 5.3 ± 1.2 years old at 7–10 days of parturition were included. All animals were fed with total mixed ration (TMR) consisted of forage (corn silage, peanut vine, rice straw) and concentrate (corn, soybean meal, wheat bran) under the stall feeding system along with free access to the fresh and clean water.

### 2.3. Estrus Detection and Ovarian Ultrasonography

Estrus behavior was observed twice a day (6:00 and 18:00) based on the presence of vaginal mucus discharge, vaginal mucosal appearance and the presence of >9 mm follicles. The real time transrectal ultrasound scanner (Desktop B-type veterinary ultrasound scanner, WED-9618-V, equipped with LV2-3/6.5 MHz rectal probe) was used to monitor ovarian follicular development. The pubertal crossbred buffalo heifers were subjected to ovarian ultrasound scanning until first ovulation and ovarian monitoring continued to consecutive ovulation. The ovarian scan in sexually mature buffaloes was performed between two consecutive ovulations. Similarly, ovarian ultrasound examination of both ovaries in the postpartum crossbred buffaloes was performed starting from day 10th after parturition until two consecutive postpartum ovulations. All experimental groups were monitored through rectal ultrasound scanner twice daily at an interval of 12 h by a single operator.

### 2.4. Follicular Dynamics

A group of small follicles with diameters of ≥4–5 mm (cohort) that appeared together was considered as follicular wave [11]. The follicles with a diameter ≥4–5 mm were monitored twice a daily over both ovaries to observe growth and regression pattern by measuring diameter. A follicle with a 10 mm diameter was considered a DF [26]. The maximum diameter (average of horizontal and vertically measured diameters) of the dominant follicle, growth rate (maximum diameter of the DF divided by number of days to attain it) and atresia rate (when follicle started to decrease in size after attaining its maximum diameter divided by duration of atresia in days) were monitored in all three groups [27,28,29,30]. The estrous cycle length was defined as period between two consecutive ovulations. Each examination was carried out by the same operator to reduce measurement error.

### 2.5. Statistical Analyses

The data were organized as Mean ± SEM and subjected to analysis of variance to study the effect of three factors (three physiology stages, different follicle waves and left- and right-side ovary). The differences between duration of estrous cycles, maximum size of dominant follicles, persistence of follicles, growth rate of dominant follicles and mean number of follicles were compared using one-way analysis of variance (ANOVA) by employing the statistical software Graph Pad Prism-6 software package (Prism-6 version 6.01, San Diego, CA, USA). The level significance was considered at *p* < 0.05 [31]. The effect of different factors (physiological status, left- or right-side ovary and follicular wave) on emergence and growth of DF, multifactor analysis of variance using R language [32,33] using following equation:

Y_ijk_ = μ + Location_i_ + Stage_j_ + Wave_k_ + eijk,

where Y indicates influencing factors, μ mean value Location_i_ indicates left- and right-side ovary, Stage_j_ indicates physiological stages (puberty, sexually mature, postpartum) and WAVE_K_ denotes first and second wave, and e_ijk_ value is random residual [34,35,36].

## 3. Results

### 3.1. Distribution of Follicular Waves in Estrous Cycle of Crossbred Buffaloes

The distribution of follicular waves in estrous cycle of crossbred buffaloes is presented in Table 1. About 89.29% pubertal buffaloes exhibited two follicular wave and 10.71% single follicular wave during estrous cycle. In the sexually mature buffaloes, there was a range of 90.90% with two and 9.09% with the three follicular wave patterns; however, one-wave follicular development was not observed. In the postpartum crossbred buffaloes, one (11.11%)-, two (77.77%)- and three (11.11%)-wave follicular patterns were observed. Overall, the two-wave follicular pattern (86.76%) was dominant in crossbred buffaloes compared with one- or three (7.35- or 5.88%)-wave follicular patterns.

### 3.2. Estrous Cycle Length and Emergence of Follicular Waves in Different Follicle Wave

The estrous length was the same in pubertal and postpartum buffaloes during one-wave estrous cycles; likewise, a similar estrous cycle duration was observed during the three-wave estrous cycle of sexually mature and postpartum buffaloes. The estrous cycle length was shorter (*p* < 0.05) in pubertal buffaloes compared with sexually mature and postpartum buffaloes during two-wave estrous cycle. The follicular emergence was on same day during the one-wave estrous cycle of pubertal and postpartum buffaloes. Additionally, the day of emergence of the first and second follicular wave was similar in pubertal, mature and postpartum buffaloes during two-wave estrous cycle. Similarly, the first, second and third follicular wave developed in a similar pattern in mature and postpartum buffaloes during the three-wave estrous cycle type (Table 2).

### 3.3. Follicular Dynamics in Pubertal Crossbred Buffalo Heifers

**Figure 1:**
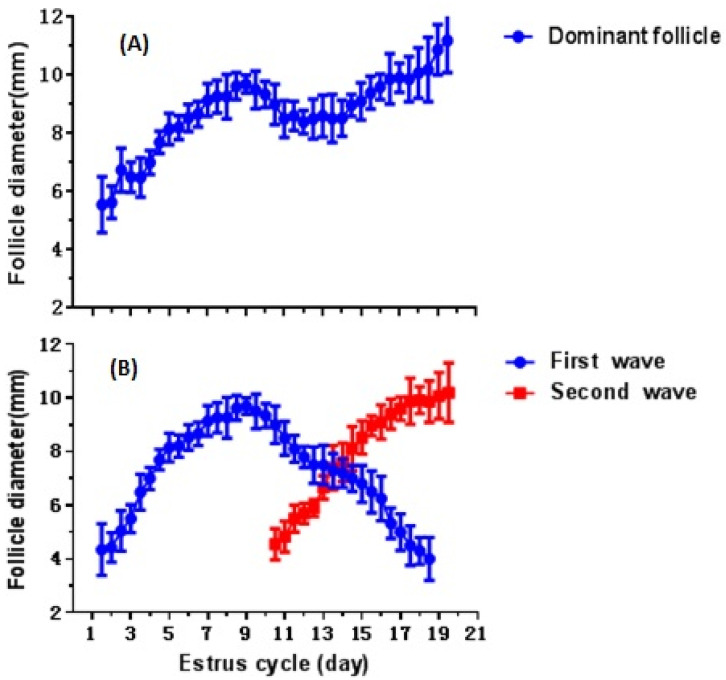
The growth profile of DF in pubertal buffaloes with a one- ((**A**), *n* = 3) and two-wave ((**B**), *n* = 25) estrous cycle.

Follicular dynamics of pubertal buffaloes are presented in Table 3 and Table 4 and Figure 1A,B. The DF developed on 1.1 ± 0.4 day of cycle, attained the peak size at day 17.1 ± 1.2 with a growth rate 0.69 ± 0.069 mm/day during the one-wave-type estrous cycle (Figure 1A). The average diameter of initial and maximum DF was similar (*p* < 0.05) across one- and two-wave patterns of estrous cycles. The maximum diameter of DF was 9.15 ± 0.50 mm irrespective of wave pattern of estrous cycle (Table 3).

The follicle dynamics during the two-wave cycle indicate the first follicle wave started on 1.5 ± 0.06 day of estrous cycle, sustained till day 10.6 ± 0.2 and became atretic after 9.5 ± 0.50 day with an atresia rate of −1.12 ± 0.14 mm/day. The second wave emerged on day 10.5 ± 0.2 of cycle and continued to grow until 18.0 ± 0.2 day of estrous cycle. The maximum follicular diameter in first and second wave was observed on day 8.6 ± 0.1 and 19.4 ± 0.2 of estrous cycle, respectively. The follicular growth rate and growth duration were significantly lower (*p* < 0.05) during one-wave as compared with the two-wave pattern of the estrous cycle. The follicle location (left or right ovary) had no significant effect on the follicular dynamics (Figure 1B, Table 4). The first ovulation occurred in pubertal buffaloes with an average body weight of 342.00 ± 22.0 kg at the age of 23.59 ± 0.4 months.

### 3.4. Follicular Dynamics in Sexually Mature Crossbred Buffaloes

**Figure 2:**
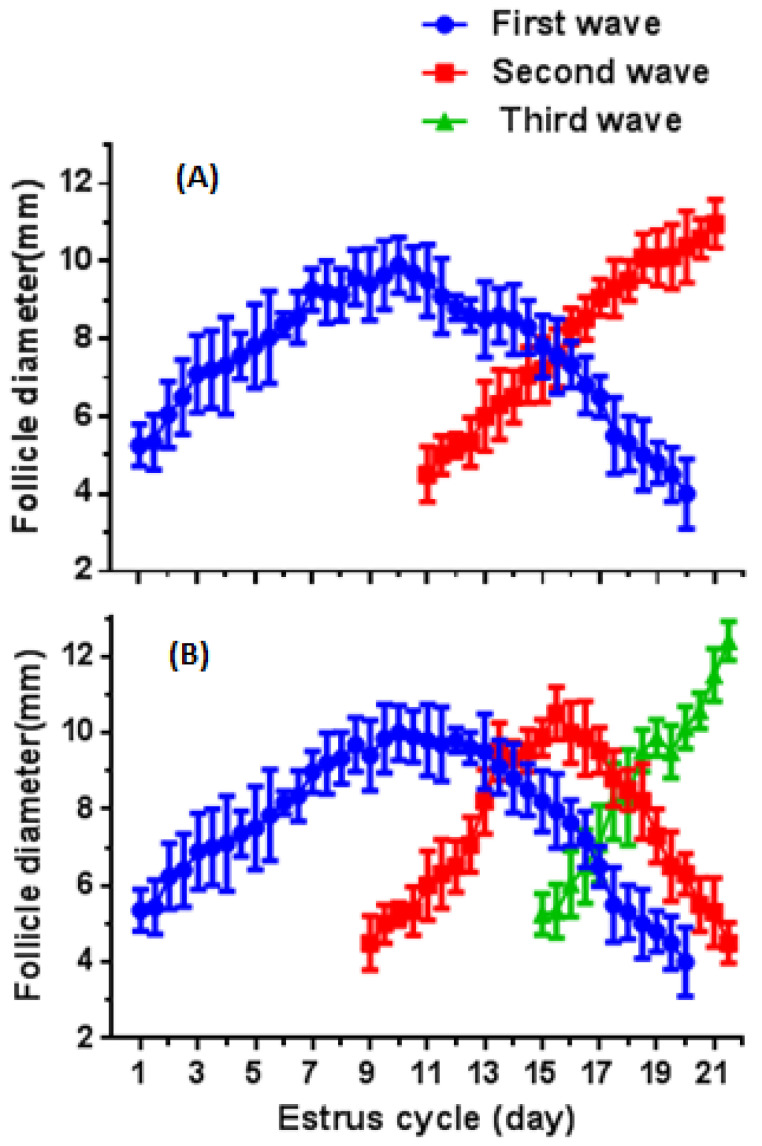
The growth profile of DF in sexually mature crossbred buffaloes with a two- ((**A**), *n* = 20) and three-wave ((**B**), *n* = 2) estrous cycle.

The initial and maximum did not vary during two- or three-wave patterns of the estrous cycle in mature buffaloes. Moreover, the growth rate and duration of growth were similar across during two- or three-wave patterns of the estrous cycle. The follicular growth rate in the second-wave pattern was 0.6 times higher than the first-wave (*p* < 0.05) pattern of the estrous cycle; however, the duration of the second follicular wave was 0.9 days less than the first follicular wave (*p* < 0.05). The average diameter of DF was 9.8 ± 0.3 mm and 10.2 ± 0.4 mm in the first and second follicular wave. The results showed that the first and second wave started on day 1.0 ± 0.02 and 11.1 ± 0.2 of cycle, respectively. The duration of first follicular wave was 9.1 ± 0.2 days, whereas it was 10.2 ± 0.4 days for the second follicular wave. The maximum follicular diameter in the first and second waves was observed on day 10.1 ± 0.2 and 21.2 ± 0.2, respectively. The location of the DF follicle (left-side ovary or right-side ovary) of sexually mature crossbred buffalo had no significant difference on follicular dynamics. The follicle atresia started on day 11.1 ± 0.50 and continued until day 20.0 ± 0.14 with rate of −0.85 ± 0.07 mm/day (Table 3, Table 4 and Figure 2A,B).

### 3.5. Follicular Dynamics in Postpartum Crossbred Buffaloes

Follicular dynamics in postpartum crossbred buffaloes are presented in Table 3. The follicular growth rate in the second wave was 0.4 times higher than the first follicle wave (*p* < 0.05) of the estrous cycle and duration of second follicle wave was 0.5 days less (*p* < 0.05) than the first follicle wave. The average diameter of DF diameter did not vary during first (10.3 ± 0.5 mm) or second (11.7 ± 0.4 mm) wave of estrous cycle. Moreover, the follicle location (left or right ovary) had no significant effect on the follicular dynamics.

**Figure 3:**
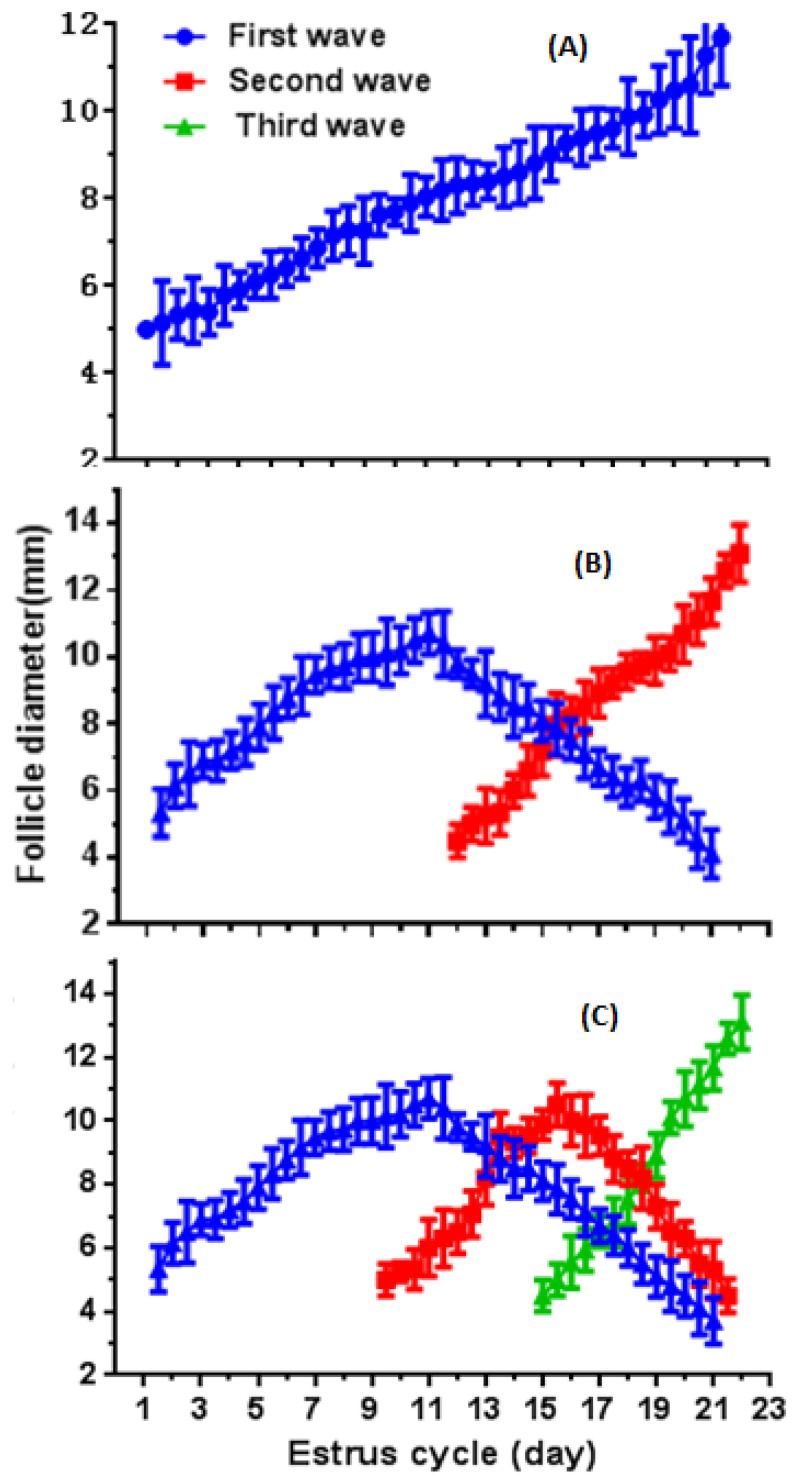
The growth profile of DF in postpartum crossbred buffaloes with one- ((**A**), *n* = 2), two- ((**B**), *n* = 14) and three-wave ((**C**), *n* = 2) estrous cycle.

The atresia rate of DF (0.71 ± 0.06 mm/day) and atresia duration (5.50 ± 0.15 days) on the right ovary have significantly higher than (*p* < 0.05) follicle atresia rate (1.12 ± 0.12 mm/day) and atresia duration (3.50 ± 0.26 days) along left side (Table 4). The onset diameter (5.3 ± 0.1 mm) and maximum diameter of DF (11.7 ± 0.4 mm) of the second follicular wave were significantly larger (*p* < 0.05) than the first follicle wave. In postpartum buffaloes with a two-wave cycle, the first and second follicular wave emerged on day 1.5 ± 0.1 and 12.1 ± 0.2, respectively, and sustained for 11.5 ± 0.2 days and 10.1 ± 0.2 days, respectively. The maximum diameters of DF in postpartum buffaloes were observed during first and second wave on day 11.0 ± 0.2 and 21.7 ± 0.2, respectively. The follicle atresia started on day 11.2 ± 0.60 and continued until day 21.0 ± 0.74 with −0.96 ± 0.08 mm/day atresia rate (Figure 3A–C; Table 4).

### 3.6. Relationship between Different Factors (Ovary Location, Physiological Stage and Follicle Wave) Effects on DF Diameter at Either the First Detection or Maximal Size and Diameter Increase in an Estrous Cycle

The results represented in Table 5 showing the relationship among the location of DF on the left or right ovary, physiological stage and number of follicle wave and effect on DF diameter at either the first detection or maximal size and diameter increase, growth rate, atresia rate in an estrus cycle. The location of the follicle and physiological effects did not affect the DF at first detection rather than the number of follicle waves. The maximum diameter of DF was influenced by the location of DF on the ovary (left ovary), physiological state (postpartum) and number of follicle waves (1st wave), respectively. There was significant effect of physiological state and number of follicle waves on increase in diameter of DF. Similar effects of physiological state and number of follicle waves were observed on the growth rate of DF. There was no significant effect of location of DF on the ovary side and number of follicle waves on atresia rate compared with physiological state. The duration of growth and duration of atresia were significantly affected by number of follicle waves compared with the location of DF on the ovary or physiological state of animals.

## 4. Discussion

Similar to other species, the follicular pattern in buffaloes is wave-like during the estrous cycle. The follicular dynamics have been described previously in pure riverine and swamp-type buffaloes in the various parts of the world such as Italy, Thailand, Pakistan, Brazil and India and reported the two follicular wave patterns predominantly in the estrous cycle [37,38,39]. In the present study, the included crossbred buffaloes belonged to the Pakistani breed (Nili-Ravi) and the local Chinese breed (Jianjhan) which were never studied for follicular dynamics in the estrous cycle. In the present study, the buffaloes were grouped in three categories to observe the impact of physiological conditions as well. The data from crossbred buffaloes of different physiological stages described how two follicular wave patterns during the estrous cycle is most common than one- or three-wave patterns. In addition to the two-wave follicular pattern, one-wave in pubertal buffaloes, three-wave pattern in sexually mature and one- or three-wave patterns of the estrous cycle were observed postpartum. Earlier reports showed two- and three-wave patterns in adult postpartum buffaloes [19,29,30,40,41,42] with few exceptions exhibited all three-wave patterns [39]. Earlier, the buffaloes were not grouped to pubertal and mature category but were listed as heifers or postpartum, and these studies showed one- to three-wave follicular pattern [42]. It is speculated that the variations in follicular wave number in cycle across the physiological conditions could be associated with managemental practices or fluctuation in LH receptors over DF results final maturation or atresia [42]. The estrous cycle length was 19.4 ± 0.4, 21.9 ± 0.4 and 21.5 ± 0.3 days in pubertal, mature and postpartum crossbred buffaloes, respectively, which depends upon emergence of follicular wave number as observed earlier [41,43]. The estrous cycle length in one-, two- and three-wave patterns corresponded to increased follicular wave numbers. Similar patterns were observed in Egyptian buffaloes where the average length of the estrous cycle was 21.75 ± 0.53 and 27.0 ± 0.58 days in two- and three-wave estrous cycle [10]. Similarly studies on Thai swamp buffaloes described that the length of the estrus cycle was 20.67 ± 1.76 days in the two-wave cycle and 23.0 ± 0.58 days in the three-wave cycle, respectively [37].

According to current data, the first follicular wave emergence was on same day in any type of wave pattern in estrous cycle. In pubertal buffaloes, the second wave mergence was earlier, which might have resulted in an earlier attainment of follicle dominance and subsequent shorter ovulatory follicle estrous cycle length compared with mature or postpartum buffaloes. The emergence of third wave in mature and postpartum the category group of buffaloes also occurred in the same day, which has been highlighted in Italian buffaloes [44]. In buffaloes with a one-wave cycle, the growth, regression and re-growth phases were shown in DF; however, two- or three-wave patterns displayed the growth and regression phases in DF, which are proceeded by the emergence of new DF of the second or third wave. The similar fashion of follicular development was observed previously in different buffalo breeds [39,42,45,46,47]. In future studies, the comparison of hormonal profile of FSH, LH and estradiol in conjunction to ultrasound monitoring of follicular turnover might be good indicator to describe the follicular pattern precisely in crossbred Chinese buffaloes.

In the present study, the first detected diameter of DF and ovulatory follicle was different in pubertal compared to postpartum buffaloes. The presently observed first detected DF diameter corroborate to previous study in heifers [47], whereas, the smaller size of DF were detected in crossbred buffaloes compared to Mehansa breed of postpartum buffaloes [31]. The current study on crossbred buffalo revealed that the maximum diameter of ovulatory follicles in postpartum, sexually matured and in pubertal buffaloes matched to earlier reports [48,49], with a few exceptions [50,51,52]. The higher growth rate was observed in pubertal buffaloes during a two-wave pattern-type cycle and this higher growth rate in a two-wave cycle of pubertal buffaloes might be related to increased sensitization of ovarian follicle pool in response to FSH and LH secretions [41,53,54]. The phases of recruitment of DF, selection and dominance are very well-characterized in breed of buffaloes [28,39]. The current study showed that first and second follicular wave emergence in estrous cycle is concordant to previous findings in water buffalo [29,55]. The interval between first and second follicular waves was shorter in pubertal as compared to sexually mature or postpartum crossbred buffaloes. This early emergence of the second follicular wave is an indication of shorter estrous cycle in pubertal buffaloes. Additionally, different hormonal profiles of buffaloes at different physiological stage could be influencing factor in pubertal buffaloes [29,30,56]. Daily average growth rate of DF in crossbred buffaloes has a similar pattern to the previous studies [29,55,57]. The overall growth rate of ovulatory follicles was greater in a two-wave cycle compared with that of a one-wave cycle in water buffalo [57]. The studies on Thai swamp buffalo revealed that the growth rate of dominant ovulatory follicles was 1.88 ± 0.39 and 1.18 ± 0.22 mm/day, respectively, during two- and three-wave cycles [36,57]. Similar reports on Indian buffalo found that the growth rate of dominant ovulatory follicles was 0.88 ± 0.17 mm/day [41].

The graphical analysis of present data demonstrated a unique finding that follicle development on the left-side ovary was higher and faster than the right side, which might be associated with compression force exerted by the vital organs located in the right region of the body [58]. Although our work involved the study of follicular dynamics on morphological basis alone, our results suggested that follicular growth in buffalo is a dynamic process, and the development of follicles occurs in waves. However, during each wave, a single follicle becomes dominant, whereas other follicles in the same wave regress. The results indicated an inter-cyclic variation in the type of follicular wave patterns in buffalo; however, this needs to be further examined during consecutive cycles in a larger group of animals along hormonal secretion and release patterns.

## 5. Conclusions

The present study revealed that Nili Ravi-Jianghan crossbred buffaloes had a two-wave follicular pattern with a duration of 20 days in the estrous cycle. There is also variation in estrous cycle characteristics and follicular dynamics with the physiological stage of buffaloes. These results highlighted the potential to improve the efficiency of fixed time artificial insemination, estrus synchronization and superovulation to increase pregnancy rate and distribution of genetic material in enhancing fertility in buffalo farming.

## Figures and Tables

**Table 1:** The distribution of follicle waves in Chinese crossbred buffaloes with different physiological stages (puberty, mature and postpartum).

Physiological Status	Follicular Waves Pattern		
	One-Wave (%)	Two-Wave (%)	Three-Wave (%)
Pubertal (*n* = 28)	3/28 (10.71)	25/28 (89.29)	-
Sexually mature (*n* = 22)	-	20/22 (90.90)	2/22 (9.09)
Postpartum (*n* = 18)	2/18 (11.11)	14/18 (77.77)	2/18 (11.11)
Overall (*n* = 68)	5/68 (7.35)	59/68 (86.76)	4/68 (5.88)

Note: The data are expressed as Mean ± SEM for different stages.

**Table 2:** Characteristics of estrous cycle of pubertal (*n* = 28), mature (*n* = 22) and postpartum (*n* = 18) Chinese crossbred buffaloes.

Characteristics	Puberty	Sexual Maturity	Postpartum
Estrous cycle duration in one-wave cycle			
DF present on right-side ovary (days)	17.3 ± 1.1	-	18.8 ± 1.9
DF present on left-side ovary (days)	16.8 ± 1.3	-	17.6 ± 1.1
Average estrous cycle duration (days)	17.1 ± 1.2	-	18.2 ± 1.6 (*n* = 2)
Estrous cycle duration in two-wave cycle			
DF present on right-side ovary (days)	19.5 ± 0.6	22.1 ± 0.5	21.8 ± 0.6
DF present on left-side ovary (days)	19.3 ± 0.5	21.1 ± 0.4	22.1 ± 0.6
Average estrous cycle duration (days)	19.4 ± 0.4 ^b^ (*n* = 25)	21.5 ± 0.3 ^a^ (*n* = 20)	21.9± 0.4 ^a^ (*n* = 14)
Estrous cycle duration in three-wave cycle			
DF present on right-side ovary (days)	-	22.3 ± 0.2	23.4 ± 0.1
DF present on left-side ovary (days)	-	21.2 ± 0.8	22.8 ± 0.5
Average estrous cycle duration (days)	-	21.8 ± 0.5 (*n* = 2)	23.1 ± 0.3 (*n* = 2)
Emergence of follicular waves during one-wave cycle			
First follicular wave (day)	1.1 ± 0.4 (*n* = 3)	-	1.5 ± 0.5 (*n* = 2)
Emergence of follicular waves during two-wave cycle			
First follicular wave(day)	1.8 ± 0.6 (*n* = 25)	1.7 ± 0.5 (*n* = 20)	1.5 ± 0.6 (*n* = 14)
Second follicular wave(day)	10.3 ± 1.4 (*n* = 25)	11.4 ± 1.3 (*n* = 20)	12.4 ± 2.3 (*n* = 14)
Emergence of follicular waves during three-wave cycle			
First follicular wave (day)	-	1.5 ± 0.5 (*n* = 2)	1.6 ± 0.4 (*n* = 2)
Second follicular wave (day)	-	9.1 ± 1.4 (*n* = 2)	9.5 ± 1.2 (*n* = 2)
Third follicular wave (day)	-	15.8 ± 1.1 (*n* = 2)	16.3 ± 1.3 (*n* = 2)

Note: The numbers are expressed as Mean ± SEM. Different small letters superscript (a,b) in the same row indicates statistically different comparisons (*p* < 0.05).

**Table 3:** The initial and maximum diameter of dominant follicle (DF) during estrous cycle of crossbred buffaloes under different physiological states (puberty; *n* = 28, maturity; *n* = 22 and postpartum; *n* = 18).

Variables	Puberty	Sexual Maturity	Postpartum
Initial diameter of DF			
On right ovary during one-wave (mm)	5.4 ± 0.2 (*n* = 15)	-	5.4 ± 0.1 (*n* = 8)
On left ovary during one-wave (mm)	5.5 ± 0.2 (*n* = 13)	-	5.2 ± 0.2 (*n* = 6)
Average initial diameter DF during one-wave (mm)	5.5 ± 0.1 (*n* = 28)	-	5.3 ± 0.1 ^b^ (*n* = 14)
On right ovary during two-wave (mm)	5.7 ± 0.2 (*n* = 15)	6.7 ± 0.2 (*n* = 7)	6.2 ± 0.3 (*n* = 8)
On left ovary during two-wave (mm)	5.8 ± 0.2 (*n* = 10)	5.8 ± 0.3 (*n* = 13)	6.1 ± 0.3 (*n* = 6)
Average initial diameter DF during two-wave (mm)	5.7 ± 0.2 (*n* = 25)	6.2 ± 0.2 (*n* = 20)	6.1 ± 0.2 ^a^ (*n* = 14)
On right ovary during three-wave (mm)	-	5.8 ± 1.1 (*n* = 2)	6.1 ± 0.9 (*n* = 2)
On left ovary during three-wave (mm)	-	6.7 ± 0.7 (*n* = 2)	6.2 ± 1.0 (*n* = 2)
Average DF during three-wave (mm)	-	6.2 ± 1.0 (*n* = 4)	6.1 ± 1.0 (*n* = 4)
Maximum diameter of DF			
On right ovary during one-wave (mm)	8.6 ± 0.3 (*n* = 12)	-	9.9 ± 0.8 (*n* = 6)
On left ovary during one-wave (mm)	9.0 ± 0.4 (*n* = 13)	-	10.7 ± 0.5 (*n* = 8)
Average maximum diameter during one-wave (mm)	8.8 ± 0.2 (*n* = 25)	-	10.3 ± 0.5 ^b^ (*n* = 14)
On right ovary during two-wave (mm)	9.6 ± 2.0 (*n* = 13)	9.5 ± 0.5 (*n* = 8)	10.9 ± 0.5 (*n* = 6)
On left ovary during two-wave (mm)	9.5 ± 0.5 (*n* = 12)	10.6 ± 0.5 (*n* = 12)	12.6 ± 0.7 (*n* = 8)
Average maximum diameter of DF during two-wave (mm)	9.5 ± 0.3 (*n* = 25)	10.2 ± 0.4 (*n* = 20)	11.7 ± 0.4 ^a^ (*n* = 14)
On right ovary during three-wave (mm)	-	11.2 ± 0.8 (*n* = 2)	11.9 ± 0.7 (*n* = 2)
On right ovary during three-wave (mm)	-	12.4 ± 0.5 (*n* = 2)	12.2 ± 0.9 (*n* = 2)
Average maximum diameter of DF during three-wave (mm)	-	11.8 ± 0.6 (*n* = 4)	12.05 ± 0.8 (*n* = 4)

Note: The numbers are expressed as Mean ± SEM. Different small letters superscript (a,b) in the same column indicates statistically different comparisons (*p* < 0.05).

**Table 4:** The growth and atresia of dominant follicle (DF) inter-estrous cycle of crossbred buffaloes during pubertal (*n* = 28), maturity (*n* = 22) and postpartum (*n* = 18) stages.

Variables	Puberty	Sexual Maturity	Postpartum
Growth rate of DF			
On right ovary during one-wave (mm/day)	0.73 ± 0.13	-	0.85 ± 0.06
On left ovary during one-wave (mm/day)	0.66 ± 0.07	-	0.92 ± 0.11
Average growth of DF during one-wave (mm/day)	0.69 ± 0.069 ^b^	-	0.68 ± 0.09
On right ovary during two-wave (mm/day)	1.06 ± 0.06	0.84 ± 0.06	0.89 ± 0.11
On left ovary during two-wave (mm/day)	1.12 ± 0.04	0.98 ± 0.05	0.98 ± 0.09
Average growth of DF during two-wave (mm/day)	1.08 ± 0.04 ^a^	0.92 ± 0.04	0.9 ± 0.07
On right ovary during three-wave (mm/day)	-	0.83 ± 0.07	0.87 ± 0.05
On left ovary during three-wave (mm/day)	-	0.89 ± 0.03	0.95 ± 0.95
Average DF growth during three-wave (mm/day)	-	0.87 ± 0.05	0.79 ± 0.08
Growth duration of DF			
On right ovary during one-wave (day)	9.05 ± 0.49	-	11.67 ± 0.31
On left ovary during one-wave (day)	9.18 ± 0.87	-	11.42 ± 0.45
Average growth duration during one-wave (day)	9.12 ± 0.47 ^a^	-	11.54 ± 0.36
On right ovary during two-wave (day)	10.45 ± 0.38	11.67 ± 0.36	11.58 ± 0.23
On left ovary during two-wave (day)	10.71 ± 0.29	10.84 ± 0.46	11.42 ± 0.36
Average growth duration during two-wave (day)	10.56 ± 0.25 ^b^	11.16 ± 0.32	11.0 ± 0.23
On right ovary during three-wave (day)	-	11.63 ± 0.29	10.93 ± 0.30
On left ovary during three-wave (day)	-	11.13 ± 0.41	11.15 ± 0.43
Average growth duration in three-wave (day)	-	11.08 ± 0.28	10.89 ± 0.34
Atresia rate of DF			
On right ovary during one-wave (mm/day)	−0.93 ± 0.06	-	−0.71 ± 0.06 ^b^
On left ovary during one-wave (mm/day)	−1.39 ± 0.32	-	−1.12 ± 0.12 ^a^
Average atresia rate in one-wave (mm/day)	−1.12 ± 0.14	-	−0.96 ± 0.08
On right ovary during second-wave (mm/day)	-	0.83 ± 0.09	0.77 ± 0.07
On left ovary during second-wave (mm/day)	-	0.96 ± 0.07	1.04 ± 0.06
Average atresia rate in second-wave (mm/day)		0.91 ± 0.08	0.93 ± 0.1
Atresia duration of DF			
On right ovary during one-wave (day)	9.52 ± 0.24	-	12.50 ± 0.15 ^a^
On left ovary during one-wave (day)	9.00 ± 0.35	-	10.50 ± 0.26 ^b^
Average atresia rate of DF during one-wave (day)	9.71 ± 0.20	-	11.50 ± 0.26
On right ovary during second-wave (day)	-	10.63 ± 0.26	11.56 ± 0.19
On left ovary during second-wave (day)	-	10.13 ± 0.22	10.31 ± 0.24
Average DF atresia duration in second-wave (day)	-	10.44 ± 0.23	10.97 ± 0.20

Note: The numbers are expressed as Mean ± SEM. Different small letters superscript (a,b) in the same column indicates statistically different comparisons (*p* < 0.05).

**Table 5:** Comparison of follicle location on ovary, physiological stage and number of follicle waves on follicular dynamics of crossbred Chinese buffaloes during estrous cycle.

Factors (Y)Parameters	Follicle Location over Ovary			Physiological State				Follicle Wave			
	Left	Right	*p*-Value	Puberty	Sexually Mature	Postpartum	*p*-Value	First	Second	Third	*p*-Value
DF diameter at first detection (mm) *	5.75 ± 0.19	6.54 ± 0.16	0.653	6.70 ± 0.16	6.03 ± 0.24	5.70 ± 0.24	0.135	5.75 ± 0.16	6.55 ± 0.17	6.21 ± 0.11	0.0003
Maximal diameter of DF (mm)	10.34 ± 0.19	9.70 ± 0.20	0.023	9.16 ± 0.20	9.89 ± 0.25	11.01 ± 0.27	0.0001	9.63 ± 00.19	10.41 ± 0.19	12.17 ± 0.12	0.003
Increase in DF diameter (mm)	4.92 ± 0.27	5.71 ± 0.25	0.076	5.79 ± 0.24	4.72 ± 0.35	5.43 ± 0.36	0.001	4.94 ± 0.24	5.68 ± 0.25	6.65 ± 0.27	0.044
Growth rate of DF (mm/day)	0.729 ± 0.12	0.766 ± 0.13	0.051	0.716 ± 0.15	0.732 ± 0.15	0.793 ± 0.16	0.0009	0.666 ± 0.13	0.828 ± 0.12	0.829 ± 0.11	0.0001
Atresia rate of DF (mm/day)	0.798 ± 0.15	0.804 ± 0.13	0.225	0.714 ± 0.13	0.797 ± 0.13	0.892 ± 0.28	0.0005	0.769 ± 0.12	0.833 ± 0.16	0.885 ± 0.17	0.648
Duration of growth (day)	9.20 ± 0.28	9.46 ± 0.29	0.519	9.30 ± 0.32	10.36 ± 0.36	11.34 ± 0.38	0.884	1.70 ± 0.27	9.97 ± 0.31	15.96 ± 0.28	0.001
Duration of atresia (day)	8.26 ± 0.24	9.95 ± 0.26	0.280	9.67 ± 0.26	10.40 ± 0.32	11.24 ± 0.36	0.168	1.61 ± 0.18	4.11 ± 0.32	-	0.0002

Note: The data were expressed as Mean ± SEM. The values (*p* < 0.05) indicate statistically difference. * DF indicate the dominant follicle.

## Data Availability

All the data and materials will be available on reasonable request from the corresponding author.
